# Diagnostic Utility of Reticulocyte Hemoglobin Equivalent in Iron-Deficiency Anemia: An Observational Study From Eastern India

**DOI:** 10.7759/cureus.101429

**Published:** 2026-01-13

**Authors:** Farah Rana, Akanksha Sinha, Minakshi Mishra, Radhika Narayan

**Affiliations:** 1 Pathology, Tata Main Hospital, Jamshedpur, IND

**Keywords:** biochemical markers, diagnostic accuracy, ferritin, inflammation, microcytic hypochromic anaemia, serum iron

## Abstract

Background

Iron deficiency anemia (IDA) continues to be a major global health concern, especially in India. Serum ferritin, a conventional marker for iron deficiency, is considered unreliable in inflammatory conditions. Reticulocyte hemoglobin equivalent (Ret-He) reflects real-time iron availability for erythropoiesis and may overcome this limitation. This study aimed to evaluate the diagnostic efficacy of Ret-He and compare it with serum ferritin in detecting IDA in patients receiving treatment at an Eastern Indian tertiary care facility.

Methods

A hospital-based observational study was conducted at Tata Main Hospital, Jamshedpur, from December 2023 to December 2024. Patients with anemia (hemoglobin <12 g/dL for females and <13 g/dL for males), ≥18 years old, were included in the study. Complete blood count parameters, including Ret-He, were measured using the Sysmex XN-1000 analyzer (Sysmex, Kobe, Japan). IDA was diagnosed based on microcytic hypochromic indices, peripheral smear findings, and serum ferritin <70 ng/mL. Receiver operating characteristic (ROC) curve analysis was done to obtain the optimal cut-off values for serum ferritin and Ret-He and their respective specificity, sensitivity, and diagnostic accuracy; negative and positive predictive values were also calculated. To compare areas under the curve (AUC), the DeLong test was employed.

Results

Among 672 patients (mean age 61.3±17.3 years, 67% females), IDA was diagnosed in 257 (38.2%) patients, including 151 (58.8%) with pure IDA and 106 (41.2%) with IDA and concomitant inflammation. In comparison to non-IDA, Ret-He was considerably lower in IDA (21.0±4.8 vs. 27.6±4.6 pg, p<0.001). ROC analysis demonstrated superior diagnostic performance of Ret-He (AUC=0.839) over serum ferritin (AUC=0.728, p=0.045). At an “optimal cut-off” of <23.6 pg, Ret-He showed 81.7% sensitivity, 73.2% specificity, and 78.4% diagnostic accuracy. Serum ferritin (<39.0 ng/ml) had higher sensitivity (90.6%) but lower specificity (50.0%). Ret-He remained low in both pure IDA and IDA with inflammation, while ferritin was markedly elevated in inflammatory states.

Conclusion

Ret-He demonstrates superior diagnostic accuracy compared to serum ferritin for identifying IDA and remains unaffected by inflammation. Its integration into routine complete blood count analysis offers a cost-effective, reliable approach for early detection of iron deficiency, making it particularly valuable in resource-limited settings and patients with concurrent inflammatory conditions.

## Introduction

Anemia affects roughly 40% of children under five, one-third of pregnant women, and 30% of non-pregnant women globally [1]. Iron deficiency, the most common cause of anemia globally, dramatically raises morbidity across all age groups [1,2]. Iron-deficiency anemia (IDA) is linked to negative impacts on cognitive or physical performance and work capacity and causes maternal and perinatal complications, highlighting the importance of early and accurate diagnosis [3].

The diagnosis of anemia is based on reduced haemoglobin concentration, while confirmation of iron deficiency traditionally relies on biochemical parameters such as serum ferritin and serum iron [4]. The acute-phase reactant serum ferritin is used to measure iron storage, but it can be artificially elevated in liver disease, infection, inflammation, and malignancy [5]. The serum iron level is also influenced by diurnal variation, dietary intake, and underlying haematological disorders, limiting its diagnostic reliability [6]. Although bone marrow iron assessment is considered the reference standard, the painful invasive nature of the sampling, cost, and subjective interpretation make it unsuitable for routine clinical practice [7].

The haemoglobin content of freshly generated red blood cells is reflected by reticulocyte haemoglobin equivalent (Ret-He), which is one of the novel parameters in recent automated hematology analyzers [8]. As reticulocytes circulate for only 1-2 days, Ret-He provides a real-time assessment of iron availability for erythropoiesis and responds rapidly to changes in iron status [9]. As a promising marker for the early identification of iron shortage, Ret-He is inexpensive, essentially unaffected by inflammatory conditions, and may be tested from the same sample that is used for complete blood count analysis [10,11].

Despite growing evidence supporting the clinical utility of Ret-He, reported diagnostic cut-off values vary widely across studies, and data from the Indian population remain limited [12], where the burden of anemia is high, and access to reliable, non-invasive diagnostic tools is essential. Therefore, this study aimed to evaluate the diagnostic performance of Ret-He in identifying IDA, compare its efficacy with serum ferritin, and determine optimal cut-off values in patients presenting to a tertiary care hospital in Eastern India.

## Materials and methods

Study design and setting

This hospital-based observational research was carried out in the Department of Pathology, Tata Main Hospital, Jamshedpur, Eastern India, over the course of 12 months, from December 2023 to December 2024. The Institutional Ethics Committee authorized the study procedure (vide reference no. TMH/IEC/NOV/105/2023 dated 29/11/2023), and before enrollment, each subject provided written informed consent.

Eligibility criteria

Individuals above 18 years presenting with anemia, defined by the World Health Organization as haemoglobin levels less than 13 g/dL in men and less than 12 g/dL in women, were selected. Patients were included if complete hematological parameters, reticulocyte hemoglobin equivalent (Ret-He), and relevant biochemical investigations were available. Exclusion criteria comprised recent blood transfusion within the preceding three months, current or recent iron therapy, pregnancy or lactation, known hemoglobinopathies or thalassemia, malignancies, and chronic liver disease.

Sample collection and laboratory methods

Samples of venous blood were drawn in an aseptic facility. Haemoglobin (Hb), reticulocyte count, MCV (mean corpuscular volume), MCH (mean corpuscular haemoglobin), MCHC (mean corpuscular haemoglobin concentration), Ret-He, and RDW (red cell distribution width) were assessed by fluorescence flow cytometry on Sysmex XN-1000 automated haematology analyzer (Sysmex Corporation, Kobe, Japan). EDTA-anticoagulated samples had been analyzed within four hours of collection. Ret-He was determined by measuring the hemoglobin content of individual reticulocytes based on forward scatter intensity after nucleic acid staining. Peripheral blood smears were prepared from EDTA samples, stained with Leishman-Giemsa stain, and examined under oil immersion by experienced pathologists to assess red cell morphology. Biochemical parameters, including folic acid, vitamin B12, serum ferritin, and serum iron, were measured from fasting venous blood samples using a Beckman Coulter AU700 photometric analyzer and DxI chemiluminescent immunoassay analyzer (Beckman Coulter, Brea, USA), following standard laboratory protocols.

Diagnostic criteria

According to the World Health Organization (WHO), low serum ferritin (<15 ng/ml in apparently healthy individuals and <70 ng/ml in individuals with infection or inflammation) signifies iron deficiency in both women and men [13]. Among our study participants, IDA was diagnosed in patients with anemia, showing microcytic hypochromic red cell indices (MCV <80 fL and/or MCH <27 pg), with peripheral smear findings consistent with iron deficiency, and serum ferritin <70 ng/mL. Since our hospital-based cohort represented unwell patients undergoing investigation, this higher ferritin threshold of <70 ng/mL was deemed appropriate to account for potential subclinical inflammation. Patients fulfilling morphological criteria for IDA but with serum ferritin ≥70 ng/mL were categorized as IDA with concomitant inflammation (IDA-inf). Remaining patients with anemia not meeting IDA criteria, including anemia of chronic disease, chronic kidney disease, megaloblastic anemia, or other causes, were classified as non-iron deficiency anemia (non-IDA).

Statistical analysis

A standardized case record form was used to capture data, and SPSS version 26.0 (IBM Corp., Armonk, USA) was used for the analysis. Continuous variables were expressed using mean ± standard deviation, whereas categorical variables were expressed using percentages and frequencies. Two groups were compared using independent sample t-tests or Chi-square tests as appropriate, and more than two groups were compared using the analysis of variance (ANOVA) test. The diagnostic effectiveness of Ret-He and serum ferritin for IDA diagnosis was evaluated using receiver operating characteristic (ROC) curve analysis. After computing the area under the curve (AUC), the optimal cut-off values were determined using the Youden index. 95% confidence intervals were used to generate the following metrics: specificity, diagnostic accuracy, sensitivity, positive predictive value, and negative predictive value. The AUCs were compared using the DeLong test. When the p-value was less than 0.05, statistical significance was determined.

## Results

The analysis comprised 672 anemic patients in all. The study population's mean age was 61.3 ± 17.3 years, and the majority of individuals (n=404, 60.1%) were older than 60. Females constituted two-thirds of the cohort (n=450, 67.0%). Moderate anemia was the most common presentation (n=336, 50.0%), closely followed by severe anemia (n=295, 43.9%) (Table 1).

**Table 1:** Distribution of study participants according to age and gender (N=672) *Mean (SD) = 61.3 (17.3) years; median (IQR) = 65 (52 – 74) years; minimum = 18 years; maximum = 93 years

Parameters	Categories	Frequency	Percentage
Age (years)*	≤40	96	14.3
	41-60	172	25.6
	61-80	333	49.6
	>80	71	10.5
Gender	Male	222	33.0
	Female	450	67.0
Severity of anemia	Mild anemia (Males: 11.0-12.9 gm/dl; Females: 11.0-11.9 gm/dl)	41	6.1
	Moderate anemia (8.0-10.9 gm/dl)	336	50.0
	Severe anemia (<8.0 gm/dl)	295	43.9

**Figure 1:**
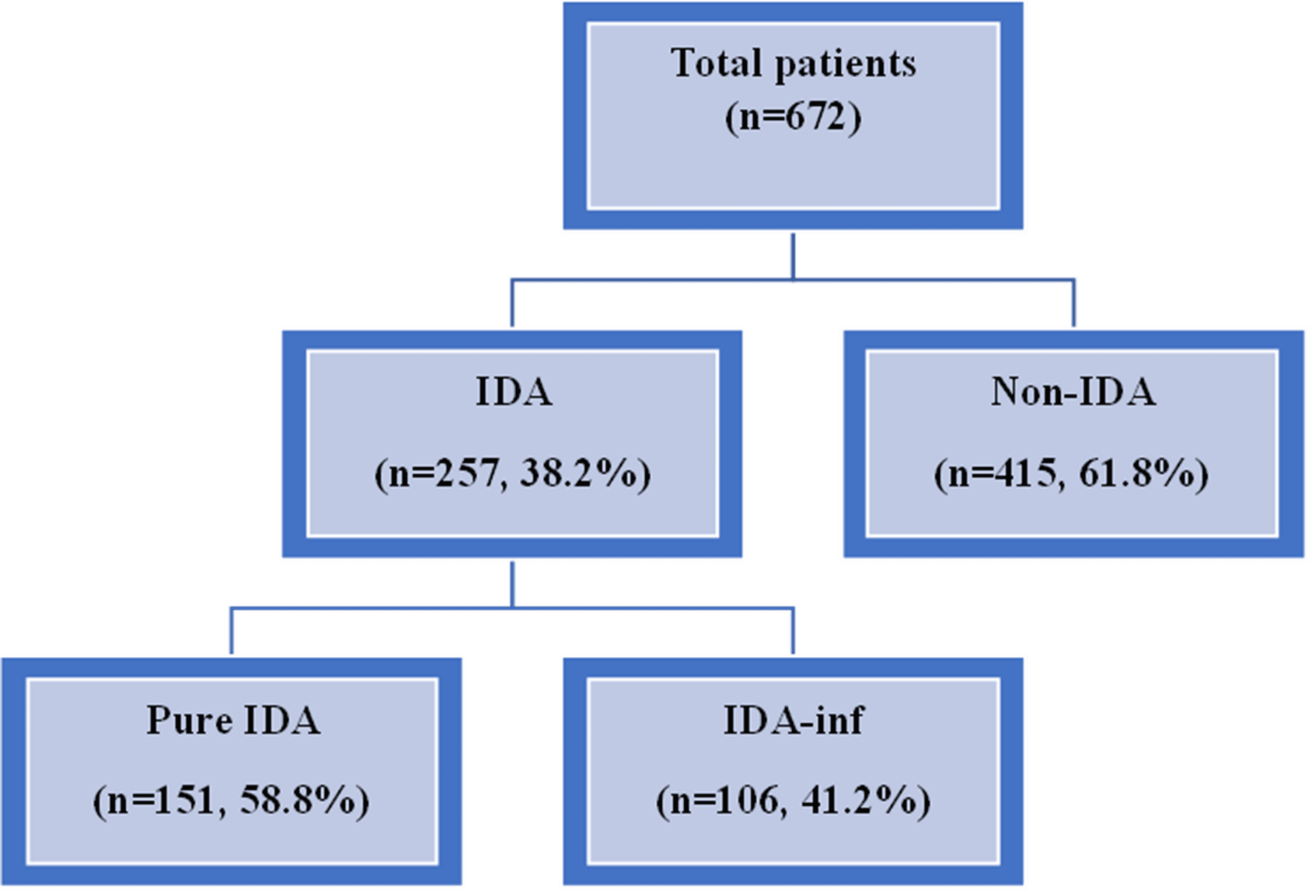
Types of anemia among the study participants IDA = iron-deficiency anemia; IDA-inf = IDA with concomitant inflammation

Based on conventional red cell indices and iron parameters, IDA was identified in 257 patients (38.2%), while 415 patients (61.8%) were classified as non-IDA. Among those with IDA, pure IDA was observed in 151 patients (58.8%), whereas 106 patients (41.2%) had IDA with concomitant inflammation (IDA-inf) (Figure 1).

Patients with IDA were significantly younger than those with non-IDA (47.5 ± 17.7 vs. 63.9 ± 16.5 years, p<0.001). IDA was more frequent among females (194/257, 75.5%) compared to non-IDA (256/415, 61.7%) (p<0.001). In comparison to the non-IDA group (8.3 ± 1.9 g/dl, p=0.005), the IDA group had lower hemoglobin levels (7.9 ± 1.8 g/dl). Patients with IDA also had significantly lower MCV, MCH, and MCHC, along with higher RDW-CV (all p<0.001). IDA patients had significantly lower Ret-He (reticulocyte hemoglobin equivalent) than non-IDA patients (21.0 ± 4.8 vs. 27.6 ± 4.6 pg, p<0.001). Serum iron concentrations were reduced in IDA (37.5 ± 34.4 μg/dl) than in non-IDA (48.8 ± 49.2 μg/dl, p=0.001). Serum ferritin, vitamin B12, and folate levels did not differ significantly between the two groups (Table 2).

**Table 2:** Comparison of demographic and laboratory parameters between study participants with IDA and non-IDA (N=672) Values are presented as mean ± SD or n (%); #p-value based on Chi-square test (for categorical variables) and independent t-test (for continuous variables); *p<0.05 was considered statistically significant Hb = hemoglobin; MCV = mean corpuscular volume; MCHC = mean corpuscular hemoglobin concentration; RDW-CV = red cell distribution width – coefficient of variation; Ret-He = reticulocyte hemoglobin equivalent; IDA = iron-deficiency anemia

Parameters	Iron-deficiency anemia (IDA) n=257	Non-iron-deficiency anemia (non-IDA) n=415	t-value/Chi-square value	p-value^#^
Age (years)	47.5 ± 17.7	63.9 ± 16.5	-4.797	<0.001*
Gender				
Males	63 (24.5)	159 (38.3)	13.662	<0.001*
Females	194 (75.5)	256 (61.7)		
Hb (gm/dl)	7.9 ± 1.8	8.3 ± 1.9	-2.758	0.005*
MCV (fL)	74.4 ± 7.9	91.3 ± 9.0	-24.842	<0.001*
MCH (pg)	22.0 ± 3.1	28.5 ± 3.2	-26.032	<0.001*
MCHC (gm/dl)	29.6 ± 2.3	31.2 ± 1.6	-11.048	<0.001*
RDW-CV	17.9 ± 3.4	16.5 ± 3.3	5.448	<0.001*
Retic count (%)	2.8 ± 5.0	2.7 ± 2.2	0.378	0.748
Ret-He (pg)	21.0 ± 4.8	27.6 ± 4.6	-17.650	<0.001*
Serum iron (μg/dl)	37.5 ± 34.4	48.8 ± 49.2	-3.204	0.001*
Serum ferritin (ng/ml)	265.5 ± 813.0	448.3 ± 871.1	-0.665	0.151
Vitamin B12 (pg/ml)	587.5 ± 550.1	683.0 ± 564.9	-1.512	0.063
Folic acid (ng/ml)	10.9 ± 6.7	12.4 ± 22.7	-1.196	0.232

On subgroup analysis, hemoglobin levels were lowest in pure IDA (7.85 ± 1.90 g/dl), followed by IDA-inf (8.07 ± 1.61 g/dl) and non-IDA (8.3 ± 1.9 g/dl) (p=0.015). Ret-He was specifically reduced in both pure IDA (20.38 ± 4.72 pg) and IDA-inf (21.86 ± 4.91 pg) compared to non-IDA (27.6 ± 4.6 pg, p<0.001). Serum iron showed a similar trend, being lowest in pure IDA and highest in non-IDA (p=0.003). In contrast, serum ferritin demonstrated wide variability, with very low levels in pure IDA (18.3 ± 17.8 ng/ml) and markedly elevated levels in IDA-inf (601.2 ± 1170.2 ng/ml) and non-IDA (448.3 ± 871.1 ng/ml) (p<0.001) (Table 3).

**Table 3:** Comparison of laboratory parameters between study participants with subtypes of IDA and non-IDA (N=672) Values are presented as mean ± SD; #p-value based on ANOVA test (for continuous variables); *p<0.05 was considered as statistically significant Ret-He = reticulocyte hemoglobin equivalent; IDA = iron-deficiency anemia; Hb = hemoglobin; IDA-inf = IDA with concomitant inflammation

Laboratory parameters	IDA n=257		Non-IDA n=415	F-value	p-value^#^
	Pure IDA n=151	IDA-inf n=106			
Hb (gm/dl)	7.85 ± 1.90	8.07 ± 1.61	8.3 ± 1.9	4.211	0.015*
Retic count (%)	2.75 ± 1.43	2.77 ± 7.66	2.7 ± 2.2	0.073	0.930
Ret-He (pg)	20.38 ± 4.72	21.86 ± 4.91	27.6 ± 4.6	160.112	<0.001*
Serum iron (μg/dl)	34.85 ± 32.14	41.19 ± 37.23	48.8 ± 49.2	5.774	0.003*
Serum ferritin (ng/ml)	18.3 ± 17.8	601.2 ± 1170.2	448.3 ± 871.1	18.756	<0.001*

**Figure 2:**
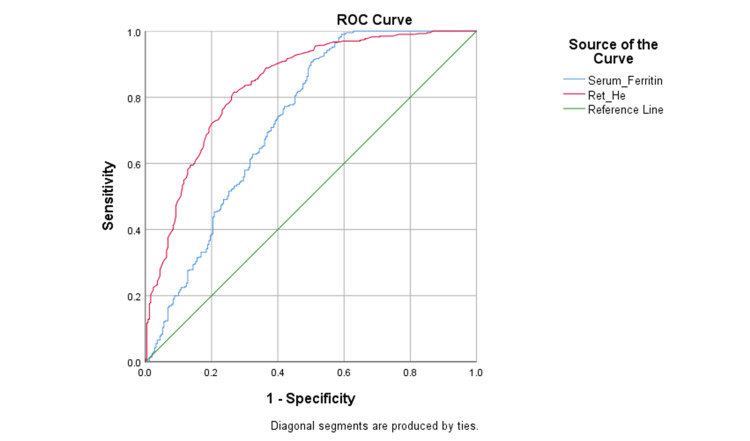
ROC curve for Ret-He and serum ferritin for the diagnosis of IDA (N=672) ROC = receiver operating characteristic curve; Ret-He = reticulocyte hemoglobin equivalent; IDA = iron-deficiency anemia

The AUC (area under the curve) for the diagnosis of IDA was 0.839 for Ret-He as well as 0.728 for serum ferritin, according to ROC analysis (Figure 2).

Overall, Ret-He demonstrated a diagnostic accuracy of 78.4%, specificity of 73.2%, with sensitivity of 81.7%, negative predictive value of 71.2%, positive predictive value of 83.1%, and a cut-off value of <23.6 pg. With a diagnostic accuracy of 74.9%, serum ferritin at a cut-off of less than 39.0 ng/ml showed greater sensitivity (90.6%) but significantly lower specificity (50.0%) (Table 4).

**Table 4:** Diagnostic test parameters of Ret-He and serum ferritin for the diagnosis of IDA (N=672) *This threshold is calculated based on the Youden index. Ret-He = reticulocyte hemoglobin equivalent; IDA = iron-deficiency anemia; PPV = positive predictive value; NPV = negative predictive value

Parameter	Cut-off value*	Sensitivity	Specificity	PPV	NPV	Diagnostic accuracy
Ret-He	< 23.6 pg	81.7 (77.6-85.3)	73.2 (67.3-78.5)	83.1 (80.0-75.4)	71.2 (66.6-75.4)	78.4 (75.1-81.5)
Serum ferritin	< 39.0 ng/ml	90.6 (87.3-93.3)	50.0 (43.6-56.4)	74.1 (71.6-76.5)	77.2 (70.8-82.5)	74.9 (71.4-78.2)

Using the DeLong test to compare the AUCs, Ret-He performed noticeably better than serum ferritin in terms of diagnosing IDA (p = 0.045).

## Discussion

Iron-deficiency anemia is often identified relatively late when relying on conventional laboratory indices such as haemoglobin, MCV, and MCH. Because mature erythrocytes have an average lifespan of approximately 120 days, alterations in these parameters tend to reflect prolonged iron deficiency rather than early disease, becoming evident only after anemia is well established. Although serum ferritin is commonly used to assess body iron stores, its diagnostic accuracy is limited in the presence of inflammation, infection, or malignancy, frequently resulting in uncertainty in routine clinical settings [5]. Reticulocyte haemoglobin equivalent's diagnostic utility was evaluated in this study, which also compared its efficacy with serum ferritin for IDA detection.

The study's main discovery was that patients with IDA had noticeably lower Ret-He values than individuals without IDA. This finding is biologically plausible, as iron-restricted erythropoiesis leads to reduced haemoglobin incorporation into newly formed reticulocytes [14]. Due to the short lifespan of reticulocytes (1-2 days), Ret-He enables a near-real-time assessment of bone marrow iron supply, unlike mature red cell indices [15]. These characteristics make Ret-He particularly useful for identifying early or functional iron deficiency.

The subgroup analysis further highlighted the clinical value of Ret-He in complex diagnostic scenarios. Patients with IDA and concomitant inflammation demonstrated markedly elevated serum ferritin levels, reflecting the acute-phase response rather than true iron sufficiency. This finding reinforces the well-recognized limitation of ferritin as an iron marker in inflammatory states [5,7]. In contrast, Ret-He values remained low in both pure IDA and IDA with inflammation, indicating that Ret-He is less affected by inflammatory processes and can reliably identify iron-restricted erythropoiesis even when conventional biochemical markers are misleading [3,10]. These findings parallel those reported by Chinudomwong et al., who demonstrated that Ret-He remained low in IDA patients with concurrent inflammation despite elevated serum ferritin levels, highlighting Ret-He's independence from inflammatory processes [6].

Receiver operating characteristic analysis demonstrated that Ret-He had superior diagnostic performance compared to serum ferritin for the identification of IDA. AUC for ferritin was substantially lower than that for Ret-He, indicating better overall discrimination. At a “cut-off value” of <23.6 pg, Ret-He showed a favourable balance between sensitivity and specificity, resulting in good diagnostic accuracy. Although serum ferritin exhibited higher sensitivity, its low specificity increased the likelihood of false positive diagnoses, particularly in patients with underlying inflammatory conditions. From a clinical perspective, the diagnostic accuracy of ferritin decreases, and it becomes less effective in diagnosing iron deficiency. This may lead to unnecessary investigations or inappropriate iron supplementation.

The optimal Ret-He cut-off identified in this study is comparable to values reported in earlier studies, although some variability exists across different populations and analytical platforms. Rao and Mirji (2024) reported a similar cutoff of 27.15 pg/cell with 57.37% sensitivity and 75.95% specificity [12], while Aedh et al. (2023) identified an optimal cutoff of 21.2 pg with 100% sensitivity and 64.1% specificity [16]. Such variation is expected due to differences in demographic characteristics, nutritional status, disease burden, and laboratory instrumentation. Notably, studies consistently demonstrate that Ret-He cutoff values in the range of 20-30 pg provide clinically useful diagnostic accuracy for iron deficiency detection [10]. The paediatric study by Poventud-Fuentes et al. reported higher cutoff values (≤30.0 pg for iron deficiency and ≤27.4 pg for IDA) with excellent diagnostic performance, suggesting that age-specific reference ranges may be necessary for optimal clinical application [17]. According to these results, setting cut-off values that are specific to a population or institution rather than implementing a global threshold may be more acceptable. Nevertheless, the consistency across studies in demonstrating lower Ret-He values in IDA supports its robustness as a diagnostic parameter.

Serum iron levels were significantly lower in patients with IDA; however, wide variability was observed, limiting its diagnostic utility. Serum iron is influenced by diurnal variation, recent dietary intake, and concurrent illness, making it an unreliable standalone marker for iron deficiency [18,19]. These limitations further support the use of functional parameters such as Ret-He, which directly reflect iron availability for erythropoiesis rather than circulating iron levels.

From a practical standpoint, the incorporation of Ret-He into routine complete blood count analysis offers several advantages. Ret-He measurement does not require additional blood sampling, adds minimal cost, and provides rapid results. These features are particularly relevant in resource-limited settings, where access to comprehensive iron studies may be restricted, and patient follow-up may be inconsistent [11,14]. Early identification of iron deficiency at the time of initial evaluation may facilitate timely intervention and reduce the morbidity associated with untreated anemia.

The study findings have significant ramifications for standard clinical practice. When ferritin interpretation is incorrect in patients with chronic inflammatory disorders, Ret-He may be a useful first-line screening measure for iron deficiency. Ret-He may serve as a valuable adjunctive screening parameter for iron deficiency, particularly when ferritin interpretation is complicated by chronic inflammatory disorders. Values below the threshold should prompt comprehensive iron studies.

Certain limitations of the present study should be acknowledged. The absence of a healthy control group precluded the establishment of reference ranges for Ret-He in individuals with normal iron status. The cross-sectional design did not allow assessment of Ret-He as a marker of response to iron therapy, an area where previous studies have demonstrated its utility. As a hospital-based study from a single tertiary care center, the findings may be subject to selection bias and may not fully represent community-based anemia patterns or primary care settings. The absence of inflammatory markers such as C-reactive protein may have resulted in potential misclassification within the IDA-inflammation subgroup, and the lack of bone marrow examination, considered the gold standard, limits definitive confirmation of iron deficiency. Additionally, unreported chronic conditions affecting iron metabolism and variability in the time from symptom onset to presentation may have contributed to unaccounted confounding.

Despite these limitations, the large sample size and inclusion of patients with diverse etiologies of anemia strengthen the validity and generalizability of the results. Future studies should focus on longitudinal evaluation of Ret-He during iron supplementation, multicenter validation with incorporation of inflammatory markers, assessment of its utility in specific populations such as pregnant women and children, and comparison with bone marrow iron assessment in a subset of patients. Nevertheless, the present study adds to the growing body of evidence supporting Ret-He as a reliable, accessible, and clinically meaningful marker for the diagnosis of iron deficiency anemia, particularly in settings where conventional markers are limited by inflammation and comorbid disease.

## Conclusions

The findings of the present study support consideration of Ret-He as a potential adjunctive marker for diagnosing and differentiating IDA from non-IDA in routine clinical practice. Compared with serum ferritin, Ret-He demonstrated superior diagnostic accuracy as an adjunctive marker and remained relatively unaffected by inflammatory processes, making it particularly useful in complementing standard iron studies when differentiating pure IDA from IDA with inflammation. A Ret-He threshold of 23.6 pg demonstrated 81.7% sensitivity, 73.2% specificity, and 78.4% diagnostic accuracy, making it a reliable parameter for ruling out IDA and guiding the need for further iron studies, though validation in diverse populations and clinical settings is warranted before widespread implementation. Its seamless integration into routine complete blood count testing, rapid availability, and cost-effectiveness can offer significant advantages over serum ferritin, especially in resource-limited settings.
